# Monitoring and Forecasting COVID-19: Heuristic Regression, Susceptible-Infected-Removed Model and, Spatial Stochastic

**DOI:** 10.3389/fams.2021.650716

**Published:** 2021-05-21

**Authors:** P.L. de Andres, L. de Andres-Bragado, L. Hoessly

**Affiliations:** 1 ICMM, Consejo Superior de Investigaciones Cientificas, Madrid, Spain; 2 Department of Biology, University of Fribourg, Fribourg, Switzerland; 3 Department of Mathematics, University of Copenhagen, Copenhagen, Denmark

**Keywords:** statistical heuristic regression, susceptible-infected-removed model, spatial stochastic, Monte-Carlo, COVID-19, SARS-CoV-2

## Abstract

The COVID-19 pandemic has had worldwide devastating effects on human lives, highlighting the need for tools to predict its development. The dynamics of such public-health threats can often be efficiently analyzed through simple models that help to make quantitative timely policy decisions. We benchmark a minimal version of a Susceptible-Infected-Removed model for infectious diseases (SIR) coupled with a simple least-squares Statistical Heuristic Regression (SHR) based on a lognormal distribution. We derive the three free parameters for both models in several cases and test them against the amount of data needed to bring accuracy in predictions. The SHR model is ≈ ±2% accurate about 20 days past the second inflexion point in the daily curve of cases, while the SIR model reaches a similar accuracy a fortnight before. All the analyzed cases assert the utility of SHR and SIR approximants as a valuable tool to forecast the disease’s evolution. Finally, we have studied simulated stochastic individual-based SIR dynamics, which yields a detailed spatial and temporal view of the disease that cannot be given by SIR or SHR methods.

## 1 Introduction

The consequences of a pandemic like COVID-19 caused by the virus SARS-CoV-2 cannot be overstated (Nature, 2021). Accurate mathematical tools allowing to monitor and forecast the evolution of the contagious disease are useful to guide social, economic and public health decisions made by governments. Nevertheless, despite the availability of powerful mathematical models (Anderson et al., 2020), initial forecasting by some organizations underestimated the evolution of the epidemics, hampering the immediate taking of necessary actions (Economist, 2020; Herzberge and Hecketsweller, 2020).

This study aims to take advantage of available worldwide data on COVID-19 (Roser et al., 2021; Dong et al., 2020) to benchmark and assign error bars to minimal models, like the susceptible-infected-recovered (SIR) with different sophistication levels (Kermack and McKendrick, 1927; Weiss, 2013; He et al., 2020a; Yang and Wang, 2020; Khan et al., 2020; Annas et al., 2020), a straightforward least-squares best-fit (LS) Statistical Heuristic Regression based on a lognormal distribution (Lam, 1988), or basic Monte-Carlo simulation (Girona, 2020; Gang, 2020). It is well-known that finding a global minimum of non-linear least-squares problems for p free parameters requires, at worst, a brute force search in p-dimensional parameter space. If each parameter can take *m* values inside a given interval, it is a non-polynomial task that scales like *m^p^* and becomes non-practical for large or moderate values of *p*. Correspondingly, there are no general polynomial bounds on the time complexity given in the number of samples and the search space dimension. These models are gauged against two variables measured daily: 1) the number of deaths, and 2) the number of new infections. Such indicators both possess advantages and disadvantages. Deaths are usually counted using a consistent methodology and, undeniably, it is an observable proportional to the spread of the disease, but the tally of deaths carry a delay of about one month on the actual dynamics of the disease. On the other hand, the number of infections is timely, but incorporates more uncertainties since it depends on details not related to the disease, e.g., on the number of tests performed. We show that the simultaneous monitoring of both observables supplemented with relatively simple mathematical approaches can be used to follow and forecast the evolution of the disease with enough accuracy to help decision-making processes and we discuss the associated error bars.

Other efforts to modeling the pandemics include sensitivity and meta-analysis to estimate averaged values for the reproduction number, incubation time, infection rate and fatality rate (He et al., 2020b), wavelet-coupled random vector functional link networks (Hazarika and Gupta, 2020), machine learning (Dhaka and Singh, 2020), and Advanced Autoregressive Integrated Moving Average Model (Singh et al., 2020). Approaches via learning algorithms are usually compared via corresponding tests (Demsar, 2006), where we recall the significant differences to statistics (Breiman, 2001).

The paper is organized as follows. In Section 2 (results and discussion), we introduce the Statistical Heuristic Regression model (SHR, section 2.1), the Susceptible-Infected-Removed model (SIR, section 2.2), and the Spatial Stochastic Individual-Based model (MC, section 2.3). After each topic, we analyze the corresponding application to different countries or regions, most notably Spain and Germany. Finally, in the section of Conclusions, we summarize and review our approaches and further discuss the application of the cases analyzed and future venues.

## 2 Results and Discussion

### 2.1 Statistical Heuristic Regression (SHR)

Epidemics can efficiently be modeled as a geometric process related to independent random events (Lam, 1988). This method yields a regression curve that describes the temporal variation of a contagious disease for the number of deaths, infections or some other relevant observable variable. Such a statistical heuristic approach results in a lognormal function,

(1) $$c_{c_{M},\mu ,\sigma }(t)=c_{M}\frac{e^{-\frac{{\left(\text{ln}\left(t-t_{0}\right)-\mu \right)}^{2}}{2\sigma^{2}}}}{\sqrt{2\pi }\sigma \left(t-t_{0}\right)}\;t-t_{0}>0,$$

which is the probability distribution function of a random variable whose logarithm, *u* = ln(*t*), is normally distributed around its mean value μ with a dispersion σ (Johnson et al., 1994). The beginning of the propagation is determined by *t*_0_ and the value of the single maximum *c_M_* = *c*(*t_M_*) happens at *t_M_* = *t*_0_ + *μ* − *σ* ^2^.

Starting from the model $\frac{\text{d}c(t)}{\text{d}t}=\alpha (t)c(t)$ and imposing general requirements on *α*(*t*) (which follow from the observed behavior of the number of daily cases) also leads to the same expression (Wenbin et al., 2013). Using entropy-related arguments, these authors have estimated that *σ* ≈ 0.4, which compares well with the averaged values for ten different western countries for deaths and infected, 0.6 ± 0.2 and 0.5 ± 0.2 respectively, cf. Tables 1 and 2. Finally, we notice that such lognormal distribution derived on (Lam, 1988) was proved useful to model the SARS outbreak in 2003 (Chan et al., 2006).

The corresponding accumulated cases are,

(2) $$c_{c_{M},\mu ,\sigma }(t)=\int_{0}^{t}c(u)\;\text{d}u=\frac{c_{M}}{2}\text{Erfc}\left(-\frac{\text{ln}(t-t_{0})-\mu }{\sqrt{2}\sigma }\right)t-t_{0}>0,$$

Given arbitrary precision, *C*(*t*) and *c*(*t*) carry the same information about the set of three parameters, *ℱ* = {*c_M_*, *μ*, *σ*}, since *c*(*t*) is simply the temporal derivative of *C*(*t*). However, in practical terms, *c*(*t*) is heavily affected by noise in the collection of the data series {*c_i_*}, and least-squares fits to the functions *C*(*t*) and *c*(*t*) are expected to determine slightly different values for *ℱ*. Therefore, we chose to report values related to *C*(*t*), which are less affected by noise. Still, we notice that the information contained in *c*(*t*) is equally valuable and sometimes simpler to obtain, in particular, the position and value of its inflexion points and single maximum.

Next, we aim to prove that the ansatz in Eqs 1, 2 reproduces the behavior of COVID-19 in ten different western countries using actual data up to the time of submission (revised version, March 2021). We observe a first wave that is relatively homogeneous among all countries (if properly normalized) and that could be considered strongly mitigated around May-June everywhere (Section Averaged profile). Other waves have later developed, which are more heterogeneous because they reflect each country’s different responses to the epidemics. A superposition of individual elementary peaks has been used to model these ulterior waves. Even if SHR merely amounts to a precise fit of the data, we observe that it carries significant advantages over the mere manipulation of the data series, {*c_i_*}, as: 1) it can be extrapolated to the near future (extrapolations should be treated with great care, but an informed extrapolation about the behavior in the future is always better than a wild guess) and, 2) it reduces long lists of numbers to an analytical expression which only depends on three parameters. Such an analytical function can be then easily manipulated to get integrals, derivatives of any order, or to search for extrema/inflexion points, etc.

#### 2.1.1 Spain

**Figure 1:**
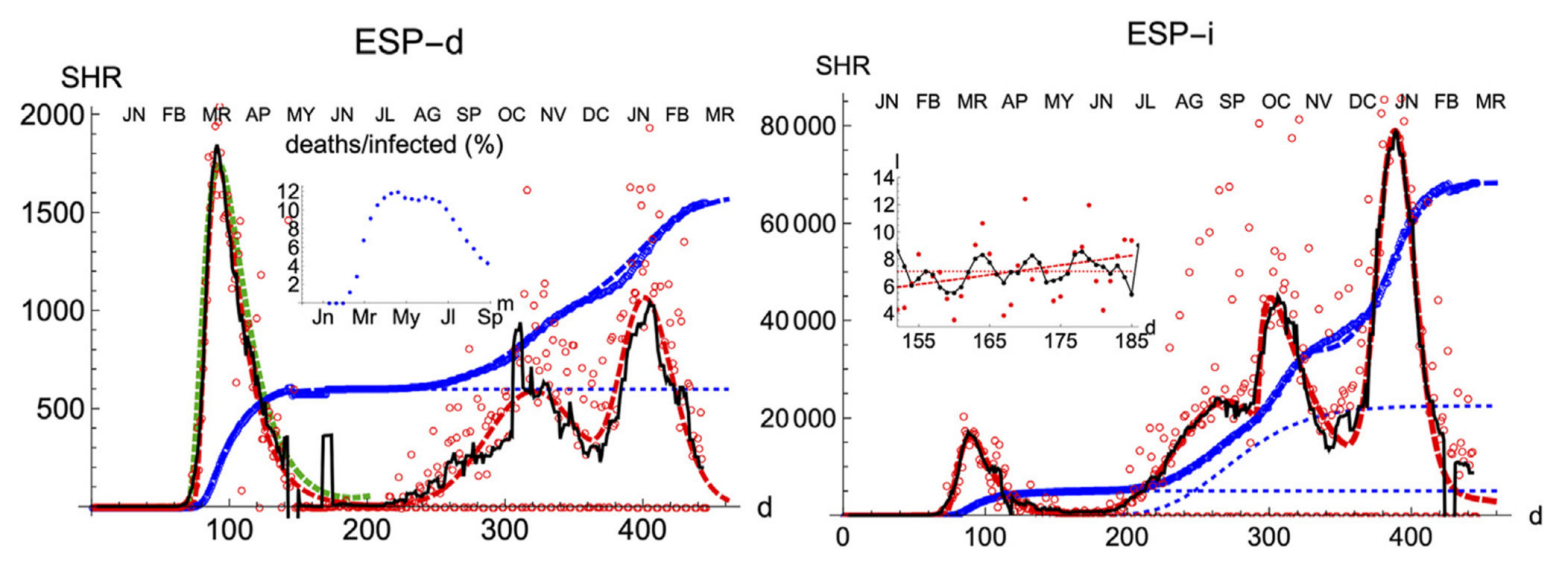
SHR/Spain. Left/Right panels: deaths/infections related to COVID-19. Data (circles) are taken from Roser et al. (2021); Dong et al. (2020). Dashed curves fit the data using Eqs 1, 2. Blue: total accumulated cases per million inhabitant. Red: daily cases per one hundred million inhabitants (the factor ×100 is introduced for the sake of better visibility on the scale of total cases only). The black thin line is a 7 days moving average of data. The green dashed line is the averaged representative curve discussed in section sct:averaged. Red and blue thin dotted lines give the contributions of individual waves. The inset (**left**) gives the percentage between deaths and infections from March to September. The inset (**right**) enlarges region II where a nearly constant number of infections takes place (red: least-squares fit to data and constant mean value. Black: 7 days moving average). Changes in data collection methodology took place on April 19th, April 25th and November 4th, producing discontinuities on the data.

Spain is a country where the disease was particularly virulent in its first wave, spreading with remarkable strength. The SHR model agrees well with the data for both deaths and infections (Figure 1 and Tables 1 and 2; a common color code is applied to facilitate comparisons: red is used for daily cases (empty dots for actual data and dashed/dotted lines for models), black for 7 days moving averages of daily cases, and blue is used for accumulated cases (again, empty dots for actual data and dashed/dotted lines for models). We have used red vs magenta for daily cases and blue vs cyan for accumulated cases to allow easy comparison between models. Other details are given in the figure captions. Together, these two variables provide a better idea of the epidemic’s course by identifying two critical items: the impact on the population via infections and, the impact on the health system via deaths. Three simple features defining the epidemics that will be rationalized later in the context of the SIR model are: 1) the exponential behavior near the origin, 2) the position and value of the single maximum in the daily number of cases and, 3) an asymmetric decay toward the future w.r.t the past. The ratio between total infections and deaths has evolved from about 1% in March to a maximum of 12% in August, but it has significantly decayed for the second wave to about 4% at the end of September (inset in left-hand side in Figure 1).

Three regions are identified in the plots, both for deaths and infections. The first region (I, wave 1) finishes approximately on the first of May 2020 (*d* = 152) and it clearly shows the three aforementioned features marking its association with an infectious disease. The second region (II, inset in right-hand side in Figure 1), goes approximately between the 150th and 200th day, and its hallmark is to sustain a fairly constant level of daily infections, *c*(*t*) = < *c_i_* >, which reflects in a linear increase of the accumulated number of cases, *C*(*t*). Region II approximately terminates near the end of the general lockdown in Spain, on the 21st of June (*d* = 173).

Neither the SHR nor the SIR models can account for a sustained period of constant infections, although they can accommodate this regime via the slowly decaying queue of the distribution where the derivative of the function is very low. In contrast, such behavior can be naturally described via MonteCarlo simulation. Likewise, while MC can describe several waves by producing more than one local maxima due to spatial inhomogeneous dynamics, SHR and SIR can only describe such a scenario via a linear combination of individual waves, each one governed with its own parameters.

Finally, in a third region (III, wave 2) the collective transmission displays again a similar behavior to the region I, marking the evolution of an out-of-control disease. The superposition of these multiple regimes, plus other waves if needed, describes the overall function well. We notice that the accuracy in the fit for any wave is not expected to be reasonably stable until at least the corresponding maximum is well developed (Section Accuracy of SHR). However such incertitude, the model predicts that in Spain the number of infections due to the second wave should be reaching its maximum in December 2020, at most in January 2021. In addition, the model predicts that the strength of the second wave is approximately weaker than the first one by a factor two, as measured by the number of accumulated certified deaths from SARS-Cov-2. Although these predictions may be affected by large error bars since the maximum in the second wave is not yet well developped, those values offer sound guidance about the course of the disease. We have used this model to extrapolate the shape of the curve by a fortnight after the last day of the corresponding available data; the resemblance to the ulterior course of the disease will be seen in the next weeks.

The accompanying number of registered infections yields a picture of the likely evolution of deaths in the following days, even if the variation in the absolute numbers from the first to the second wave is dominated by the change in the number of tests performed. Given the large dispersion of raw data due to difficulties to collect them it is clear the necessity to perform moving averages and the advantages of working with least-square approximants that can be extrapolated a few days ahead, a statement that is true for the behavior of other countries. While deaths only show two waves so far, infections identify at least four local maxima that can be correlated with different events, like the end of the summer vacations or the occurrence of several bank holidays in Spain where the population has been moving and mixing in great numbers.

#### 2.1.2 Germany

Compared with other countries with large populations, Germany has managed the pandemics quite well, as it is observed by comparing the number of cases per inhabitant. Moreover, its evolution has been recorded with consistency both for deaths and infections. Therefore, it is an appropriate benchmark for any model.

**Figure 2:**
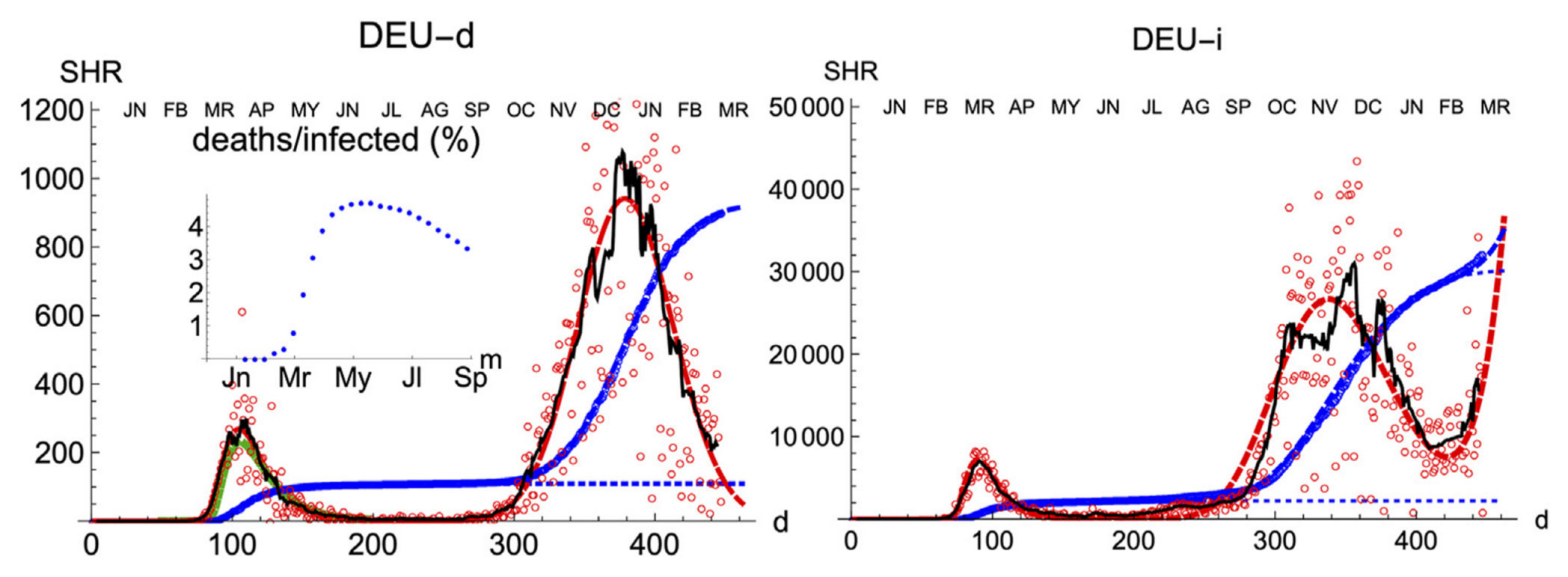
SHR/Germany. Symbols as in Figure 1.

Similarly to Spain, the SHR model can be used to accurately represent the disease evolution using only three parameters per wave (Figure 2). Curiously enough, best-fit values for μ and σ are quite similar to Spain (Tables 1, 2), indicating that, independently of the absolute strength, there are common underlying features in both cases. Therefore, it is interesting to explore the ability of a *single normalized averaged* curve to represent such contrasting cases as Spain and Germany, using *C*(∞) ∝ *c_M_* as the single only free parameter. Such a curve is represented in Figures 1, 2 by the green dashed line having *μ* = 3.53 and *σ* = 0.56 (Section Averaged profile), and it is clear that despite having such a limited freedom for fitting (since it only depends on one parameter), it provides a very reasonable approximation to the data. In contrast to Spain, the ratio between total infections and deaths in Germany evolved from about 1% in April to 4% in September, which is about three times lower than for Spain (inset in Figure 2).

#### 2.1.3 Other Countries

**Figure 3:**
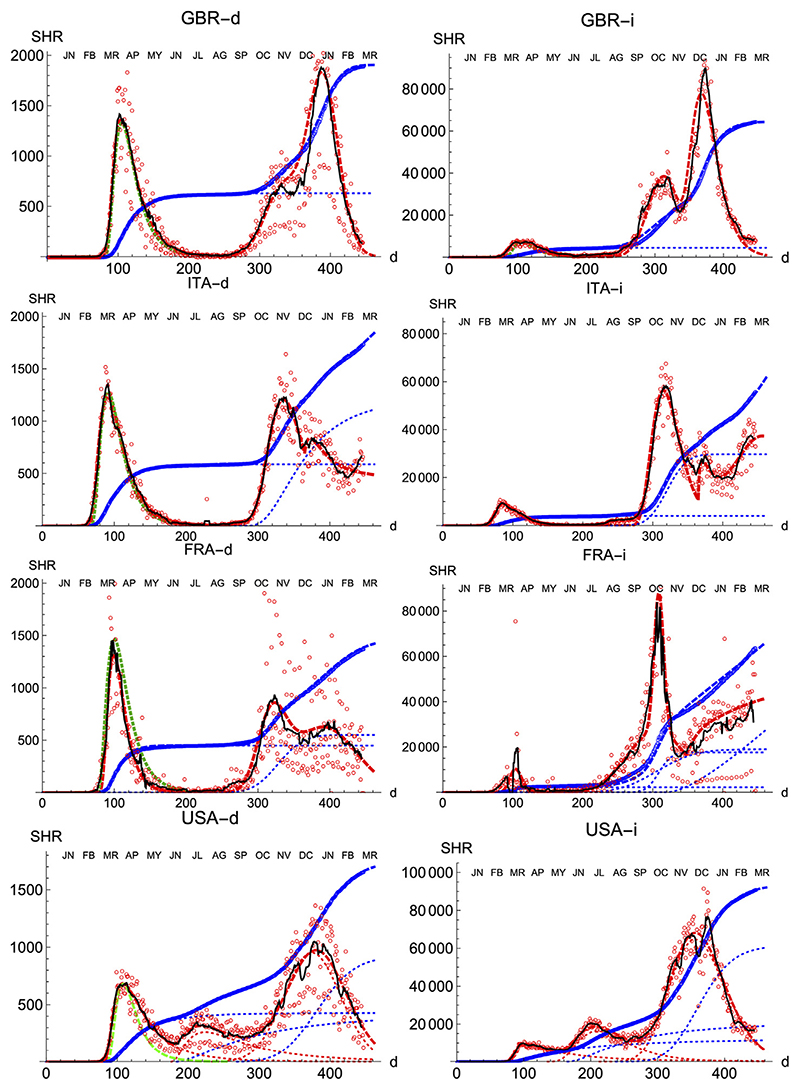
SHR/Other countries (I). Symbols as in Figure 1. Changes in methodology took place in United Kingdom (GRB) on May 20th and July 3rd, and in France (FRA) on May 28th.

**Figure 4:**
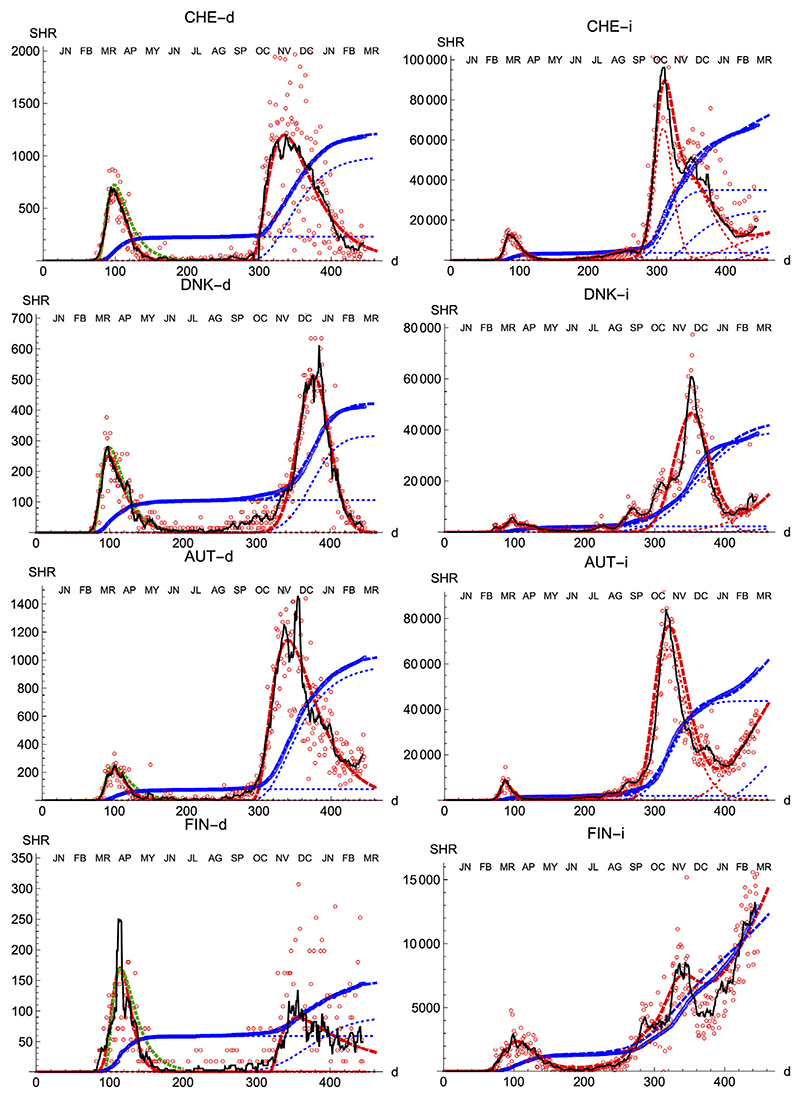
SHR/Other countries (II). Symbols as in Figure 1.

We also prove the capabilities and versatility of the SHR ansatz to reproduce the observed data by applying the same methodology to a pool of western countries: Great Britain (GBR), Italy (ITA), United States (United States), France (FRA), Switzerland (CHE), Denmark (DNK), Austria (AUT) and Finland (FIN), cf. Figures 3, 4. In general, the agreement is quite good, both for deaths and infections. Among other advantages, this procedure allows a quick and simple monitoring of the evolution of the disease in the different countries. In particular, it is a useful tool to identify and forecast the appearance of a second wave. At the moment of writing, only the United States has fully developed the maximum associated with the second wave and, from the combined behavior of deaths and infections, it could be argued that the country is clearly heading toward a third wave. Since this is the only case so far, it is not possible to characterize well such a second wave by a proper average of different countries, although it seems fair to say that it is represented by a wider distribution of daily deaths, (e.g. the second component represented by the dotted red curve corresponds to having *μ* = 5.5 and *σ* = 1.2) and a lower value at the peak by about a factor 2.

#### 2.1.4 Regions: NYC vs Madrid

**Figure 5:**
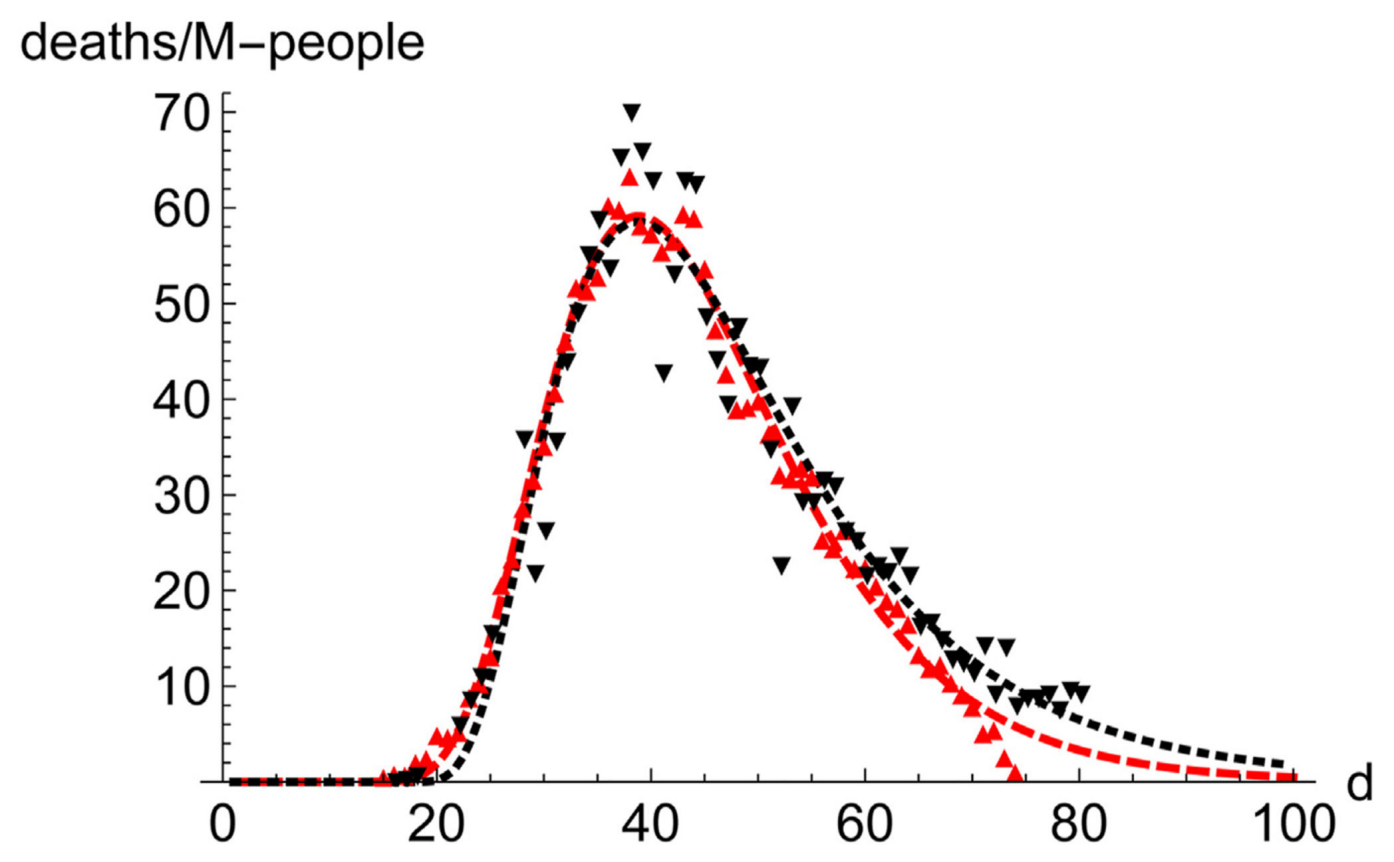
Comparison of the evolution of number of deaths in NYC and the region of Madrid (CAM) during the first wave. NYC (9.1 M people, red, down pointing triangles, ▽, dashed line) and Madrid (6.7 M people, black, upwards pointing triangles, △, dotted line) during the first wave. The data and SHR fits for both locations were juxtaposed matching the day with the maximum number of deaths, aiming to highlight the similarities. The values of the CAM were also scaled to the ratio of population between the two regions $\frac{9.1}{7.6}$ to enable a better comparison.

Prominent places where the infection spreads quickly are densely populated regions, which constitute the core of the propagation of the disease. Therefore, it is interesting to compare the distribution of cases in those regions. We have juxtaposed the performance of New York City (9.1 M-people, NYC) and the Community of Madrid (6.7 M-people, CAM) in the first wave (Figure 5). To highlight the similarities rather than the differences they are superimposed in such a way that the position (day) of the maximum coincides. Furthermore, CAM has been scaled by the ratio of respective populations, which makes the value at the maximum very similar for both regions. Despite all the differences between these regions, it is clear that a typical pattern emerges, which leads us to investigate the advantages of working with averages.

#### 2.1.5 Averaged Profile

**Figure 6:**
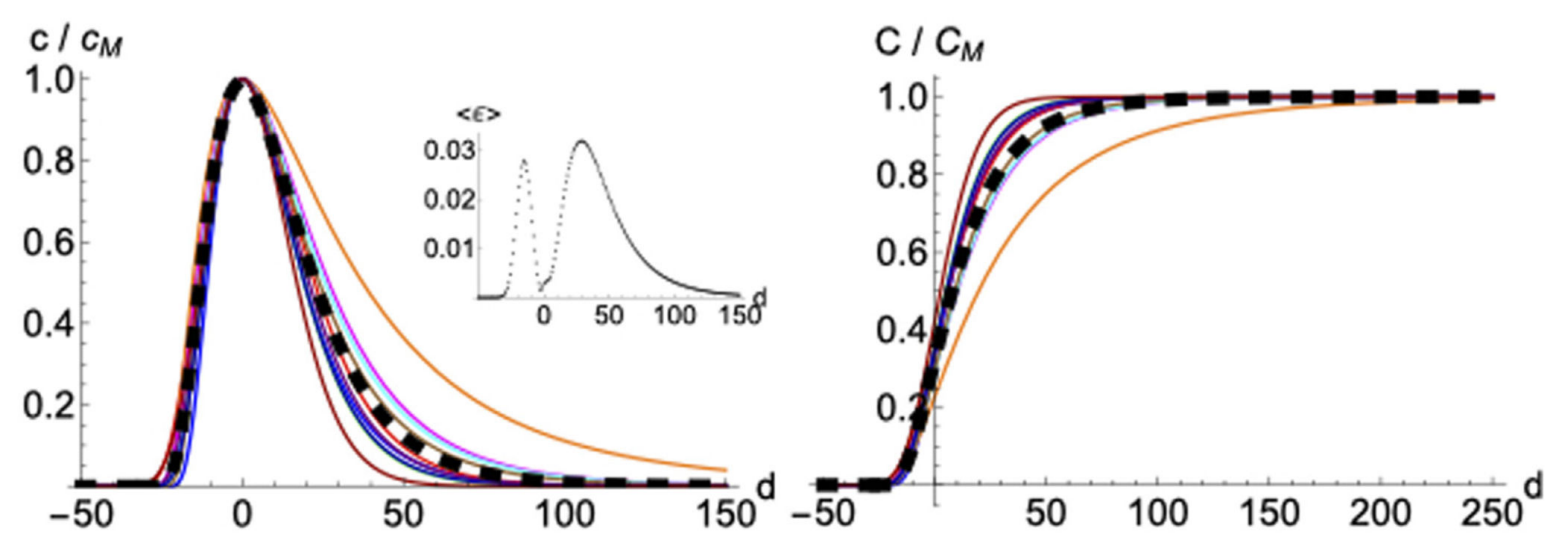
Averaged profile. Daily *c*(*t*) (left panel) and accumulated *C*(*t*) = *r*(*t*) cases (right panel)for ten different countries, normalized to its maximum value and displaced rigidly in time so *C*″ (*t*) = *c*′ (*t*) = 0 the same day. Color codes are: (1) Spain (blue), (2) Germany (red), (3) France (green), (4) United States (orange), (5) Italy (magenta), (6) Great Britain (cyan), (7) Switzerland (purple), (8) Denmark (brown), (9) Austria (darker blue) (10) Finland (darker red). The black thick dashed line gives the average over the ten countries, with *μ* = 3.53, *σ* = 0.56.

Normalizing and superimposing the curves for COVID-19 deceases on different countries such that the maximum in *c*(*t*) is in a common position (*t_M_*) allows us to focus on similarities. Despite slight differences, nine out of the ten arbitrarily chosen western countries are all well represented by a normalized average function, 〈*c*(*t*)〉, (Figure 6). United States shows as an outlier; a warning about the quite different boundary conditions from the other European countries. Since second waves are not fully developed (except in United States) it is not possible yet to ascertain if such a universal average could represent faithfully second waves, even if maybe with different effective values of μ and σ owing to the different boundary conditions that may apply. We have not tried the same procedure with the infected because of the greater temporal and spatial variability of procedures used to define that variable. However, results for deaths in the first waves make us believe that such a representative average could also be applied to a properly defined observable for infections. Excluding United States, the maximum average error made by substituting the actual data by the average function (ϵ = *c*(*t*) − < *c*(*t*) >) is ≈ 0.03 in units of *c_M_*, which happens near the inflections points where the function *c*(*t*) has decayed to ≈ 0.4 (see inset in left panel in Figure 6). Therefore, the averaged curve yields an answer with a fractional error of ≈ ± 5%, which is an excellent initial guess taking into account that it only depends on a single parameter, *c_M_*. Such parameter *c_M_* can be easily obtained from a single point: the maximum value in the daily distribution of cases for each wave, which we derive from a moving average of a few days (seven days makes appropriate averages that account for regular weekly routines and removes most of the noise for all the cases we have analyzed).

#### 2.1.6 Accuracy of SHR

To be able to confidently use a least-squares statistical regression to a given data set {*C_i_*} ((*i* = 1, *n*)) the main question is how many data points, *n*, are needed to yield a reasonable estimation of the evolution of the epidemics based solely on the extrapolation of the fitted functions. Such question is relevant considering how unreliable extrapolations usually are (Press et al., 2007). Indeed, any simple algorithm to forecast the evolution of an epidemics can only be valuable if reasonable error bars can be assigned to predictions.

**Figure 7:**
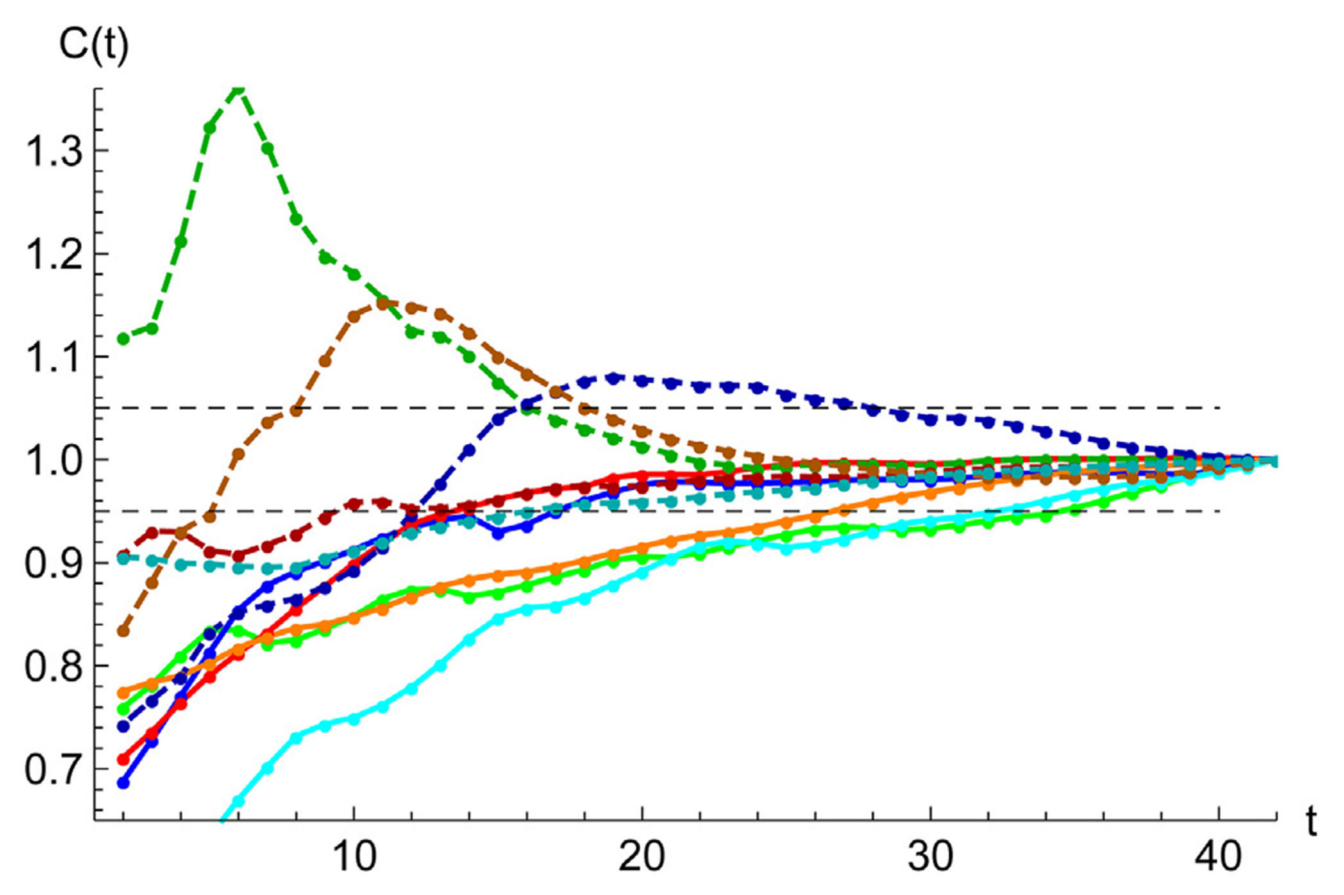
Accuracy of SHR best fits. Starting at the second inflection point, t_2_, fractional error in the evolution of the predicted accumulated number of cases *C*(*t*) for: (1) GBR (green), (1) ESP (blue), (2) ITA (red), (3) GBR (green), (4) FRA (orange), (5) United States (cyan), (6) CHE (dashed darker green), (7) DNK (dashed darker blue), (8) DEU (dashed darker red), (9) AUT (dashed darker orange) (10) FIN (dashed darker cyan). The region ± 5% is delimited by black dashed lines. A common normalizacion has been used by making *C*(*t*_2_ + 40) = 1 for all cases.

A simple target to quantify the error is to study the behavior of the expected total number of cases, *C*(∞), as a function of *n*. Figure 7 shows the variation of the predicted asymptotic value as a function of the available amount of data after the second inflexion point. In most cases, a fractional accuracy of ±15% is achieved a fortnight after the second inflexion point, which is further decreased to ±5% in another fortnight.

### 2.2 Susceptible-Infected-Removed (SIR)

So far, we have shown that SHR qualifies as a quick and straightforward way to describe the evolution of an infectious disease. If adequately used, i.e., attached with appropriate error bars, it can be extrapolated to make predictions in the near future, since the functional forms associated with Eqs 1, 2 adapt so well to the observed data.

However, a better understanding of the dynamics of the epidemics can be obtained from a set of differential equations which describe its time evolution. The simplest model for the evolution of a contagious disease is to postulate that the rate of new infections is proportional to the number of infected people itself, $\frac{dI(t)}{dt}=\frac{I(t)}{\tau_{0}}$, which results in an unbound exponential growth, $I(t)\propto e^{\frac{t}{\tau_{0}}}$, and makes a characteristic mark for the onset of a pandemic.

Such a simple model does not take into account how the rate of infections decreases as the number of infections approaches the total population. Therefore, a refined version is to divide a given population of size *N* into three classes (*𝒮*, *ℐ*, *ℛ*): 1) susceptible entities who can catch the disease, *S*(*t*), 2) infected ones who have the disease and transmit it, *I*(*t*), and 3) removed ones who have been isolated, died, or recovered and become immune, and are therefore not able to propagate the disease, *R*(*t*). In this model, individuals pass from the susceptible class *S* to the infective class *I* and finally to the removed class *R* with rates determined by a set of ordinary differential equations (ODEs) (Kermack and McKendrick, 1927; Anderson, 1991; Hethcote, 2000; Weiss, 2013). The ODEs derive from the interactions of the entities in the different classes, which can be represented as

𝒮+ℐ→2ℐ,ℐ→ℛ,

where we assume generalized mass-action kinetics (Müller and Regensburger, 2012) (with slightly different scaling with respect to *N*). First, it is assumed that the number of susceptible individuals decreases at a rate proportional to the density of infected, $i(t)=\frac{I(t)}{N}$ times the number of susceptible individuals, *S*(*t*),

(3) $$\frac{\text{d}S(t)}{\text{d}t}=-\frac{{\left(S(t)\right)}^{n}}{\tau_{0}}i(t),$$

where *τ*_0_ is an adjustable parameter that represents a typical time to transmit the disease, and *n* is a parameter that influences the ability of the disease to infect susceptible individuals in a nonlinear way, (e.g. it might represent the effect of the viral load). Its main effect is to alter the temporal scale of the epidemics, which in some circumstances facilitates the fitting of the model to real data. The standard SIR model is recovered with *n* = 1.

Removed entities originate from infected; therefore, its variation is assumed to be proportional to the number of infected,

(4) $$\frac{\text{d}R}{\text{d}t}=\frac{\left(I(t)\right)}{\tau_{1}},$$

where *τ*_1_ is an adjustable parameter that represents a typical time to recover from the disease. This equation merely helps to count the total number of removed from the beginning of the infection up to a given day *t*,

(5) $$R(t)=\int_{0}^{t}\frac{\left(I(u)\right)}{\tau_{1}}\text{d}u.$$

Lastly, the infected vary according to the inflow of susceptible individuals who become infected minus the outflow of infected that have been removed,

(6) $$\frac{\text{d}I}{\text{d}t}=\frac{{\left(S(t)\right)}^{n}}{\tau_{0}}\;i(t)-\frac{I(t)}{\tau_{1}},$$

The derivative $\frac{dI}{dt}$ moves from positive to negative depending on the balance between both terms in the equation and it determines a single peak in *I*(*t*) (for *n* = 1, *I*′ (*t*) = 0 for $\frac{S(t)}{N}=\frac{\tau_{0}}{\tau 1})$.

The task at hand for a given population of *N* elements is to determine the parameters, *τ*_0_, *τ*_1_ and *n*, that best reproduce the behavior of the epidemics by solving the coupled system of differential Eqs 3, 6, subject to some initial conditions, e.g., *S*(0) = *N* − 1, *I*(0) = 1. Good agreement with data can be used to lend an interpretative value to *τ*_0_ and *τ*_1_ (unlike parameters μ and σ which only have a statistical meaning). The ratio ${ℜ}_{0}=\frac{\tau_{1}}{\tau_{0}}$ is called the effective reproductive number; values ℜ_0_ ≫ 1 characterize a virulent disease where *R*(∞) = *S*(0).

First, we focus on the task of simulating a population where $s(0)=\frac{S(0)}{N}=r(\infty )=\frac{R(\infty )}{N}=1$. For this particular case, ℜ_0_ ≫ 1 and the entire susceptible population is removed at the end. The proposed algorithm goes as follows.

1) We use the daily number of deaths to identify the position and maximum value in the infections/deaths data: $t_{M}^{*}$ and $i_{M}^{*}$.
2) *τ*_0_ is the main parameter that determines the position of the peak in *i*(*t*). We estimate a value for *τ*_0_ that brings the maximum in *i*(*t*) near $t_{M}^{⋆}$.
3) We get an approximate value for *τ*_1_ from the expression $i_{M}^{*}=1-\frac{1}{{ℜ}_{0}}\left(1+\text{ln}\;{ℜ}_{0}\right)$ (Weiss, 2013).
4) The value $i_{M}^{⋆}$ yields *N* in the particular case of *R* (∞) = *N* = *c_M_*. We adjust the value of *N* to agree with $i_{M}^{*}$.
5) We minimize the root-mean square deviation, $\frac{\chi }{N}=\sqrt{\frac{1}{n}\sum_{i=1}^{n}{\left(C_{i}-R\left(t_{i}\right)\right)}^{2}}$, between the number of accumulated cases predicted by the model, *R*(*t*), and the recorded data, *C_i_*, to find optimal values for *τ*_0_, and *τ*_1_.

#### 2.2.1 Spain

**Figure 8:**
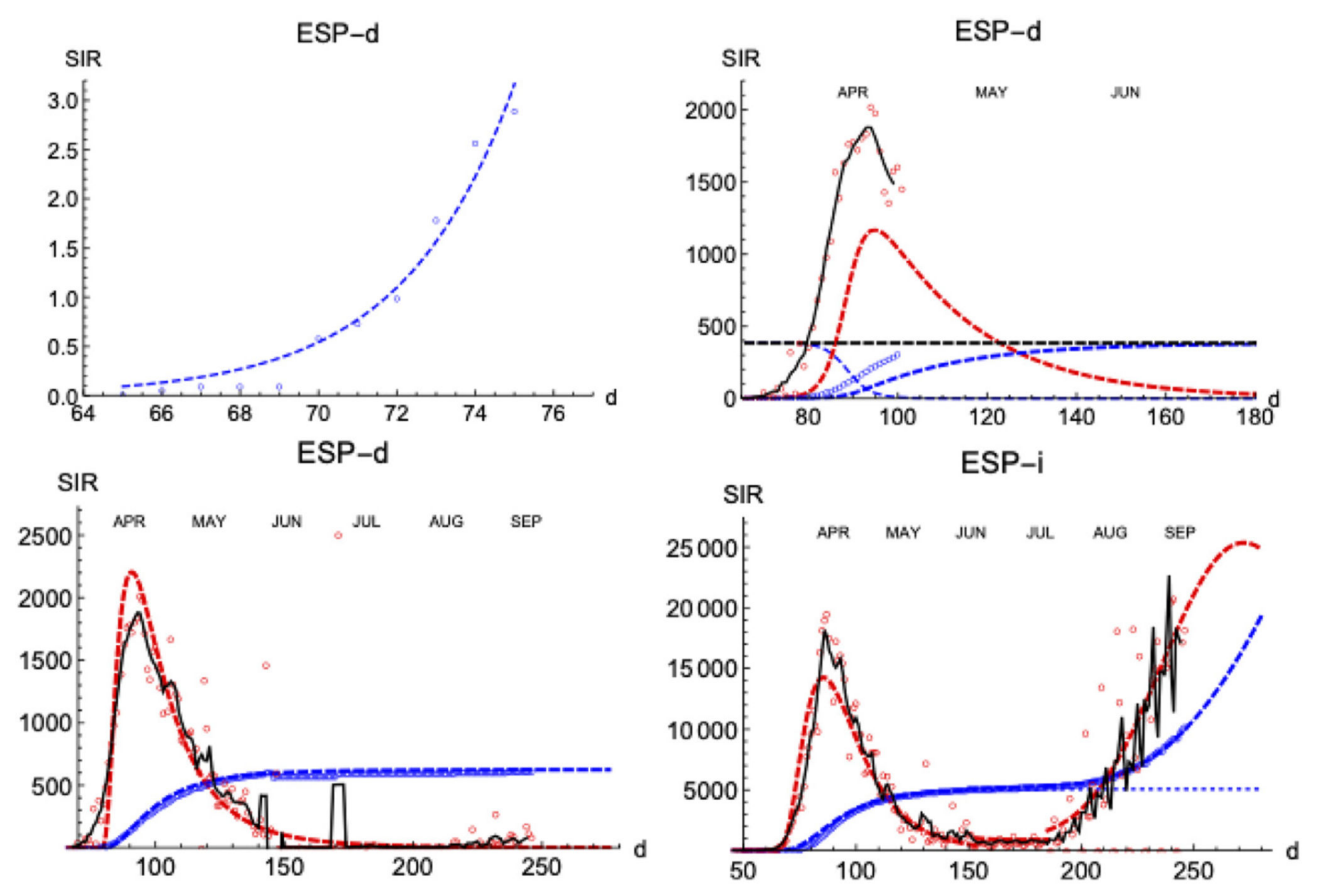
SIR/Spain. Left upper panel: exponential fit near the onset. Right upper panel: initial iteration for deaths (see text). Left lower panel: final iterations for deaths. Right lower panel: final iterations for infections. Blue: accumulated cases, *R*(*t*) (per million people). Red: daily cases, *I*(*t*) (×100 to increase visibility in the same scale as *R*). Black is a 7 days moving average of data to help the eye.

Similarly to the SHR analysis we have presented above, we illustrate the performance of the SIR model by first looking at the distribution of deaths and infections in Spain (Figure 8). The lower left panel shows how numerical solutions to SIR equations match very well the temporal behavior of the epidemics under the condition *s*(0) = *r* (∞) = 1 for optimized values of *τ*_0_ and *τ*_1_ (Table 3). Dispersion of data in the daily reported cases is usually smaller before the peak is reached (the quasi-exponential region) and fluctuations grow in importance after the maximum is reached–which is a general observation holding for most of the countries we have studied. We assign it to the balance between different *currents* transferring individuals between the three classes, the phenomenon responsible for the appearance of a single maximum in daily cases for a given wave in the pandemics.

The proposed procedure works for the deaths subset as follows. First, the curve of daily cases is followed up to the appearance of its maximum, which to circumvent the noise is identified from a smoothed curve obtained by a five-day moving average, $I_{M}^{*}$($t_{M}^{*}$ = 96) = 17.1 per million people (the single daily maximum value is *I_M_*(*t_M_* = 94) = 20.2). The SHR model for accumulated deaths using only data up to six days past the maximum yields a prediction of total deaths of *N* = 383, which is off the final mark by about 40%.

Once the maximum is identified, the quasi-exponential behavior near the origin is used to estimate an initial value for *τ*_0_ (Supplementary Eq S4). For Spain, the first case happens at *t* = 65, and the first inflexion point is at *t*_1_ = 82. Therefore, the first 10 points (about halfway to *t*_1_) are used to get an exponential fit to the accumulated number of cases that yields *τ*_0_ ≈ 2.8 ± 0.3. Such a value, combined with an initial guess ℜ_0_ = 10 produces a maximum in the curve of daily deaths at *t_M_* = 103. Accordingly, *τ*_0_ is decreased until we locate the maximum closer to the right position. For *τ*_0_ = 2.2 we get *t_M_* = 95 and *I_M_* = 11.7 (per million inhabitant). Therefore, we update the value of N using the ratio $\frac{17.1}{11.7}$ and start an efficient local Levenberg-Marquardt minimization of the root-mean-squared deviation between the actual data and the computed values. This is done to simultaneously optimize *N*, *τ*_0_ and *τ*_1_ (Figure 8, left-lower panel). Taking into account that only data up to six days past the maximum have been used, it is remarkable that this self-consistent procedure reduces the fractional error between the prediction of the SIR model and the data from 40% to ±3%, being the root-mean-squared deviation (RMSD) between the accumulated data and the predicted function $\frac{\chi }{N}=0.6\%$. Such a low RMSD value matches the good visual agreement observed. We believe that the logic behind the steps proposed above amounts to more than a recipe to get a best fit, yielding meaning to the values obtained and their interpretation.

Next, we explore how the SIR model represents the evolution of the number of infections. The number of infections is a magnitude that carries larger error bars, but it can provide timely information on the evolution of the epidemics (Figure 8, right-lower panel shows the case for Spain). As expected, infections start earlier than the deaths (*t* = 32 vs *t* = 65), but need more time to attain its maximum value (*t* = 54 vs *t* = 25 after the first case).

A prominent feature is the existence of the second wave of infections separated from the first one by a region of *sustained constant* number of cases, as we have discussed in Figure 1. To fit the data, we superpose the two waves, each with its own defining parameters. However, the constant region between waves cannot be easily accommodated in these models and it is a clear indication of a different stage in the epidemics with low but sustained transmission of the disease at a pace similar to the one at which individuals are removed (while in the SIR model usually it is assumed that *τ*_1_ > *τ*_0_). We shall come back to this point in the context of Monte-Carlo simulation. Finally, we notice that this second wave of infections has finally overlapped with a third one, as it is noticeable in Figure 1.

#### 2.2.2 Germany

**Figure 9:**
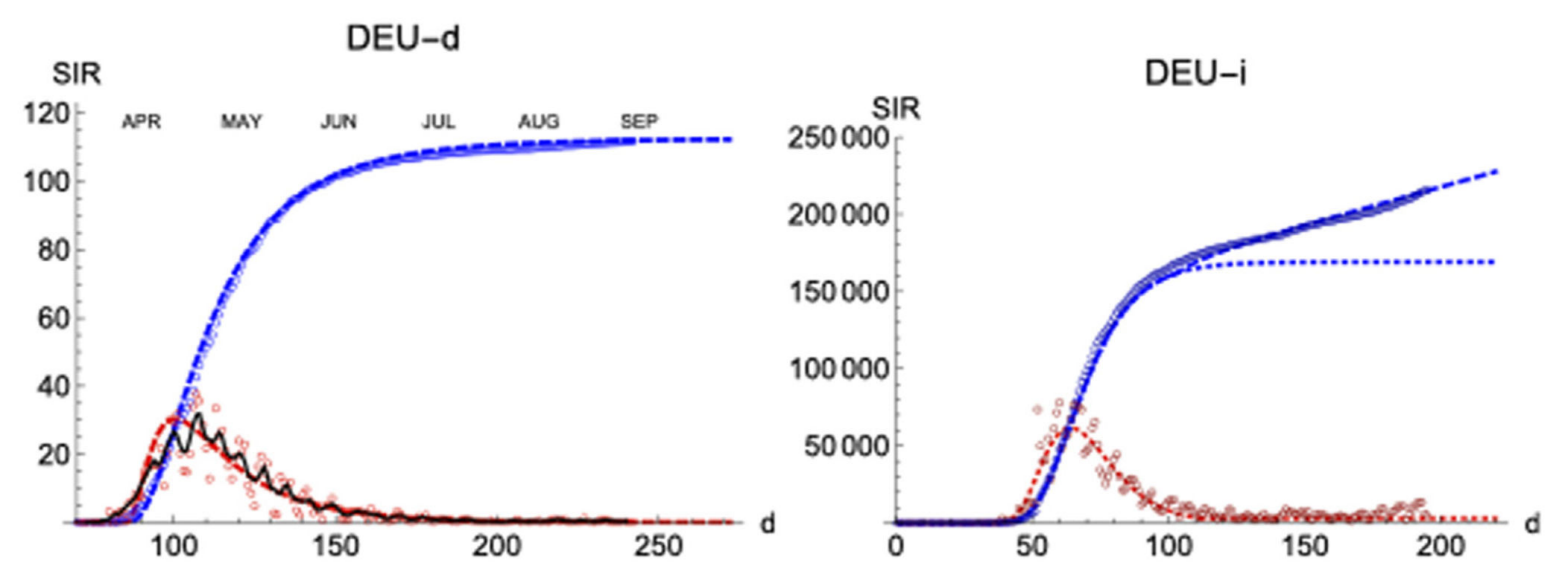
SIR/Germany. Left/Right panels: Deaths/Infections. Blue: total cases, *r*(*t*). Red: daily cases, *i*(*t*) (×10). Other symbols as in Figure 8.

We have applied the same procedure to Germany, a country which had in the first wave about four times less casualties per million inhabitant than Spain. The left panel of Figure 9 shows the final iteration for the daily and accumulated number of deaths, which again predicts the total number due to the first wave with accuracy ≈ ±3% of the final true value, even if we have only used data up to six days past the maximum. The RMSD between the accumulated data and the predicted function is $\frac{\chi }{N}=0.5\%$, which reflects the good visual agreement observed too.

Regarding the infections, it is interesting that the region of sustained infection is also observed, although a second wave is only weakly apparent up to the present day (t = 200). Again, infections start earlier (*t* = 28) w. r.t deaths (*t* = 70). Furthermore, maximum values are attained after a longer amount of time (*t* = 63 for infections (*t* = 63) than for deaths (*t* = 30), counted after the first case, following a similar procedure to the one for Spain.

#### 2.2.3 Other Countries

**Figure 10:**
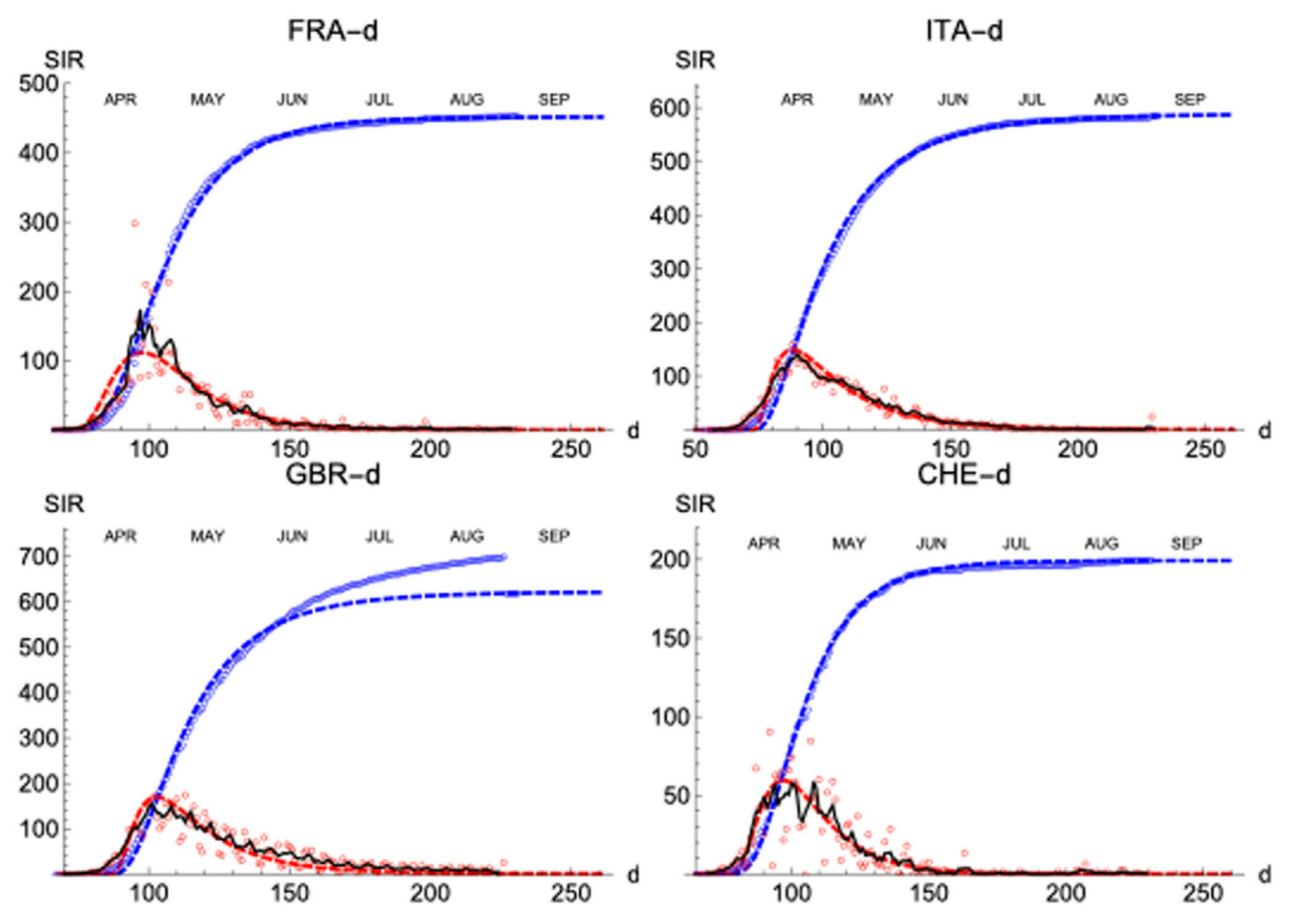
SIR (deaths)/FRA, ITA, GBR, and CHE. Respectively left to right and top to bottom. Symbols and lines as in Figure 8.

Finally, similar results have been observed in four more countries: France, Italy, Great Britain and Switzerland (Figure 10; Table 3).

### 2.3 Spatial Individual-Based Model

To gain further insight into the spatio-temporal evolution of COVID-19, we consider next a stochastic discrete-time individual-based model in which the spread propagates on a two-dimensional *N* × *N* lattice, where each node represents an individual. The dynamics are Markovian, and we will use Monte Carlo (MC) to sample from its distributions in time, which is a technique known to handle well difficult collective effects in many-body systems, like e.g. the magnetic phase transition in the 2D Izing model (Peliti, 2011). The *N* ^2^ individuals can be in any of the three states of the SIR model, making transitions between them with two probabilities: 1) for someone susceptible to be infected *𝒮* → *ℐ*, *p_i_* and, 2) for someone infected to recover and be removed *ℐ* → *ℛ*, *p_r_*. At each time-step, individuals make transitions between classes according to the corresponding probabilities. We consider various scenarios of uniform and spatially dependent Markov dynamics.

**Figure 11:**
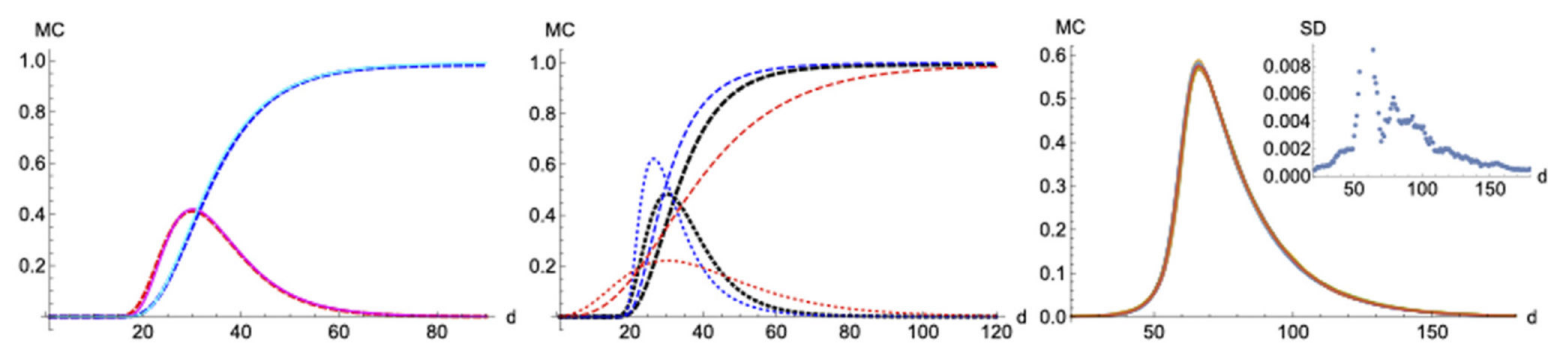
Monte-Carlo simulations. Left panel: MC (continous) vs SIR (dashed). *N* = 10000. Red/Magenta: infected. Blue/Cyan: removed. Parameters used in MC are: *p_i_* = 0.5*i(t), *p_r_* = 0.1. Parameters used in SIR are: τ_0_ = 2, τ_1_ = 8. Middle panel: various MC scenarios for infected (dotted) and removed (dashed). Thick black: probabilities as in left panel. Blue: nearest-next neighbors increased probability of infection increases to *p_i_* = 0.75 *i*(*t*). Red: both probabilitiesfor infection and removal are kept constant values, *p_i_* = 0.1 and *p_r_* = 0.05. Right panel: average and standard deviation (inset) of 10 random realizations for *p_i_* = 0.2*i(t) (double for nearest-neighbors infections) and, *p_r_* = 0.05.

First, we start with a single isolated case of infection per 10^4^ individuals, and we use $p_{i}(t)=\frac{i(t)}{\tau_{0}}$ for *𝒮* → *ℐ* in close analogy to the SIR model, while we assign a constant value $p_{r}=\frac{1}{\tau_{1}}$ to the second transition probability, *ℐ* → *ℛ*. Comparing MC simulations for *N* = 100 with $p_{i}(t)=\frac{1}{2}i(t)$ and $p_{r}=\frac{1}{10}$ to the deterministic SIR with *τ*_0_ = 2.1 and *τ*_1_ = 9 yields an excellent agreement between both approaches for same initial values, which confirms the adequacy of Monte-Carlo techniques (left panel in Figure 11, where both results cannot be distinguished). By way of example, we modify the model to increase the probability of infection of individuals in next-neighbors contact with members already infected to $p_{i}=\frac{3}{4}i(t)$. As expected, infections grow faster near the onset, the daily maximum happens earlier and results in a larger and narrower peak (while keeping the final total number the same, Figure 11 blue dotted line compared to thick dotted line).

**Figure 12:**
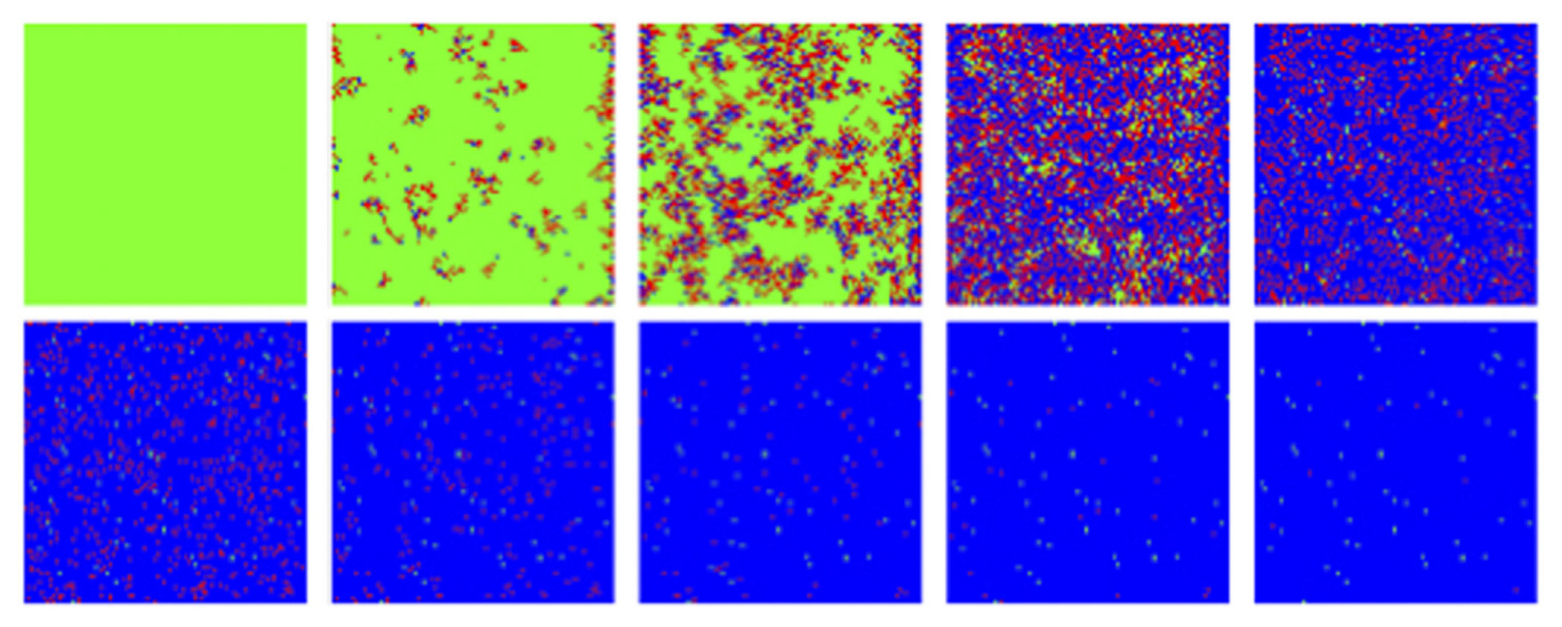
MC/Spatial. Typical evolution of individuals (pixels) with MC steps (10 steps between frames). Green, red and blue correspond to susceptible, infected and recovered. Other parameters are: *N* = 100, *p_i_0__* = 0.001, *p_i_1__* = 0.1, *p_r_* = 0.05.

On the other hand, a scenario where the infection probability is kept constant (*p_i_* = 0.1, *p_r_* = 0.05) results in a wider and smaller maximum (the infection and recover constant probabilities have been adjusted to yield the peak near the same MC steps on the previous cases, Figure 11 red dotted line compared to thick dotted line). For these conditions, a typical temporal evolution of individuals (pixels) is shown in Figure 12. A weak tendency to clustering is observed, although the system is seen to reach a quasi-homogeneous state fast.

Unlike SIR, this model can sustain in a natural way a constant background of infections if at some point in the epidemics *p_i_* becomes very similar to *p_r_*, establishing a transient regime which we categorize as qualitatively different from the region where the daily distribution derived from SHR or SIR is simply too low. This is a feature that can be observed in real data (Figure 1 inset in the right-hand side).

Finally, we checked how statistical properties of the model perform and scale under different lattice sizes and parameters via simulation. The distributions over time for *N* = 100 and *N* = 1000 are virtually indistinguishable as long as the initial infectious individuals are kept in the same ratio. In order to further visualize the stochasticity under the chosen scale, we show in the right panel of Figure 11 ten randomly chosen realizations out of one hundred runs with random initial positions of infectious in the lattice. As the starting day where the infection expands is random, we have rigidly displaced the time of the samples such that they peak on the same day. Then, the ten different realizations and their averaged value lie nicely on the same curve and the standard deviation displayed in the inset is seen to be acceptably small.

## Conclusion And Future Venues

We have analyzed and compared three mathematical approaches of increasing complexity to investigate the dynamics of COVID-19. A take-home message is that all three approaches have enough flexibility to embody the pandemics’ actual behavior for ten arbitrarily chosen countries. However, they display different error bars and have different abilities to be extrapolated into the future to produce valuable predictions.

We have proved that a least-squares SHR-model based on the lognormal distribution is well suited to describe the epidemic’s evolution using only two free parameters per infection wave. Confronted against real data up to the second inflexion point, the values determined for these parameters are well converged and stable, guaranteeing fractional error bars of ±5%. Therefore, the SHR-model is suitable to extrapolate tendencies to the next one or two weeks, even in the presence of noisy data. A simpler averaged version depending only on a single free parameter per wave has been shown to be adequate to be used as a first approximation, albeit with larger associated incertitudes. We have also considered a generalized deterministic SIR dynamics to analyze the temporal evolution of the disease. In this case, the corresponding two free parameters are well converged and stable once the maximum in the daily distribution of cases is passed, i.e. about a fortnight before the SHR reaches a similar accuracy. Besides the two deterministic models, we have considered stochastic individual-based dynamics reflecting the daily changes in individuals’ classes. We examined both the case of uniform and neighbour-dependent transitions via a Monte-Carlo simulation, which has an excellent correspondence with the analogue SIR model’s temporal evolution.

While such simple dynamics ignore individual, spatial or further inhomogeneities (e.g., genetic, socioeconomic, or other differences) we have proven that they can reproduce, predict and forecast relevant features of the actual COVID-19 dynamics. In particular, they provide reasonably robust ways to monitor and forecast the actual temporal evolution of contagious diseases in different environments, while only requiring basic mathematical tools.

The analysis of ten different countries makes us conclude that the SHR model can be extrapolated into the future with at most a 5% fractional error after a fortnight passed the second inflexion point. On the other hand, the SIR model, which includes two free parameters only too, seems more stable and can be used with a similar accuracy about one week passed the maximum. Finally, the MC model is helpful to study the interactions between separated regions developing the epidemics.

By comparing SHR and SIR we find an excellent correlation between functions *c_σ_*,*_μ_*(*t*) and *i*_*τ*_0__,_*τ*_1__ (*t*), and their respective cumulative distributions *C*(*t*) and *r*(*t*), which suggest that an analytical parametrized solution for SIR might be possible by trying a variational-like approach:

$$i_{\tau_{0},\tau_{1}}(t)≔c_{\sigma ,\mu }(t)+\delta (t).$$

Our results strongly suggest that useful bounds can be found for *δ*(*t*). Such promising venue will be explored in the future.

On the other hand, the excellent agreement between SIR and MC (provided the transition probabilities are chosen in accordance with the hypothesis behind the SIR model) opens new prospects to whrite spatially resolved SIR-like models that might be solved applying Markov chains techniques.

## Supplementary Material

The Supplementary Material for this article can be found online at: https://www.frontiersin.org/articles/10.3389/fams.2021.650716/full#supplementary-material

SI

## Figures and Tables

**Table 1:** Parameters for SHR model (confirmed deaths, first wave). *p*: country’s population (millions). μ and σ: parameters in the lognormal distribution. *C*(∞): asymptotic value for accumulated cases (per million person). *R* ^2^ and *r* ^2^ : R-squared correlation factors for *C*(*t*) and *c*(*t*), respectively. *t_M_* and *t*_2_ : maximum and second inflection point (origin is the first of January 2020).

Country	^*p*^	^μ^	σ	C(∞)	_R_2	d_1_	t_M_	t_2_
G. Britain	66.65	3.61 ± 0.01	0.76 ± 0.02	688 ± 4	0.9999	07/03	37	52
Spain	46.94	3.55 ± 0.06	0.43 ± 0.03	606 ± 2	0.9997	05/03	28	40
Italy	60.36	3.75 ± 0.02	0.51 ± 0.01	581 ± 1	0.9999	23/02	37	52
United States	327.20	3.95 ± 0.01	1.08 ± 0.04	456 ± 5	0.9997	01/03	47	64
France	67.00	3.27 ± 0.03	0.59 ± 0.02	451 ± 1	0.9998	15/02	53	63
Switzerland	10.23	3.77 ± 0.03	0.36 ± 0.02	199 ± 1	0.9999	06/03	32	45
Germany	83.02	3.71 ± 0.02	0.45 ± 0.01	110 ± 0.3	0.9999	09/03	36	50
Denmark	5.81	3.60 ± 0.02	0.53 ± 0.01	106 ± 0.4	0.9999	16/03	23	36
Austria	8.86	3.30 ± 0.04	0.62 ± 0.03	80.5 ± 0.2	0.9997	13/03	26	38
Finland	5.52	4.31 ± 0.14	0.20 ± 0.03	59.6 ± 0.3	0.9995	22/03	33	45

**Table 2:** Parameters for SHR model (confirmed infections, first wave). *p*: country’s population (millions). μ and σ: parameters in the lognormal distribution. *C*(∞): asymptotic value for accumulated cases (per million person). *R* ^2^ and *r* ^2^ : R-squared correlation factors for *C*(*t*) and *c*(*t*), respectively. *t_M_* and *t*_2_ : maximum and second inflection point (origin is the first of January 2020).

Country	^*p*^	^μ^	σ	C(∞)	R^2^	d_1_	t_M_	t_2_
G. Britain	66.65	4.09 ± 0.01	0.47 ± 0.02	4511 ± 11	0.9999	31/01	78	99
Spain	46.94	3.55 ± 0.06	0.43 ± 0.03	5082 ± 14	0.9996	1/02	56	67
Italy	60.36	3.75 ± 0.02	0.51 ± 0.01	4083 ± 9	0.9999	31/01	56	71
France	67.00	3.66 ± 0.03	0.40 ± 0.02	2743 ± 12	0.9998	25/01	68	80
United States	327.20	4.23 ± 0.04	0.93 ± 0.03	13300 ± 400	0.9391	21/01	49	107
Switzerland	10.23	3.79 ± 0.03	0.35 ± 0.02	4028 ± 5	0.9999	26/02	31	41
Germany	83.02	3.84 ± 0.02	0.38 ± 0.01	2038 ± 10	0.9999	27/01	63	23
Denmark	5.81	4.35 ± 0.12	0.28 ± 0.04	2056 ± 30	0.9994	27/02	42	61
Austria	8.86	2.92 ± 0.05	0.57 ± 0.03	2334 ± 7	0.9996	26/02	29	37
Finland	5.52	4.41 ± 0.06	0.29 ± 0.02	1341 ± 12	0.9998	30/01	74	96

**Table 3:** Parameters for SIR model (first wave). *N*(number of individuals), τ0 and τ_1_ (given in days). Upper: deaths per million people. Lower: infections per million people.

Country	*N*	*τ*_0_	*τ*_1_
Spain	622	1.81	18.23
G. Britain	621	2.57	22.47
Italy	586	2.47	25.59
France	454	3.68	19.41
Switzerland	200	2.97	16.70
Germany	112	2.48	23.15
Country	*N*	*τ*_0_	*τ*_1_
Spain	5,082	3.11	18.05
Germany	2,111	3.49	12.50

## Data Availability

The original contributions presented in the study are included in the article/Supplementary Material, further inquiries can be directed to the corresponding author.

## References

[R1] Anderson R (1991). The Kermack-McKendrick epidemic threshold theorem. Bull Math Biol.

[R2] Anderson RM, Heesterbeek H, Klinkenberg D, Hollingsworth TD (2020). How will country-based mitigation measures influence the course of the COVID-19 epidemic?. The Lancet.

[R3] Annas S, Isbar Pratama M, Rifandi M, Sanusi W, Side S (2020). Stability analysis and numerical simulation of seir model for pandemic covid-19 spread in indonesia. Chaos, Solitons and Fractals.

[R4] Breiman L (2001). Statistical Modeling: The Two Cultures (with comments and a rejoinder by the author). Statist Sci.

[R5] Chan JSK, Yu PLH, Lam Y, Ho APK (2006). Modelling sars data using threshold geometric process. Statist Med.

[R6] Demsar J (2006). Statistical comparisons of classifiers over multiple data sets. J Machine Learn Res.

[R7] Dhaka A, Singh P Comparative analysis of epidemic alert system using machine learning for dengue and chikungunya.

[R8] Dong E, Du H, Gardner L (2020). An interactive web-based dashboard to track COVID-19 in real time. Lancet Infect Dis.

[R9] Economist (2020). Forecasting covid-19-a terrible toll. The Economist.

[R10] Gang X (2020). A novel monte carlo simulation procedure for modelling covid-19 spread over time. Scientific Rep.

[R11] Girona T (2020). Confinement time required to avoid a quick rebound of covid-19: Predictions from a monte carlo stochastic model. Front Phys.

[R12] He J, Chen G, Jiang Y, Jin R, Shortridge A, Agusti S (2020a). Comparative infection modeling and control of covid-19 transmission patterns in china, south korea, italy and iran. Sci Total Environ.

[R13] He W, Yi GY, Zhu Y (2020b). Estimation of the basic reproduction number, average incubation time, asymptomatic infection rate, and case fatality rate for COVID-19: Meta-analysis and sensitivity analysis. J Med Virol.

[R14] Herzberge N, Hecketsweller C (2020). Les modèles déboussoles pour prédire l’évolution de l’épidémie due au coronavirus.

[R15] Hethcote HW (2000). The mathematics of infectious diseases. SIAM Rev.

[R16] Johnson NL, Kotz S, Balakrishnan N (1994). Continous Univariate Distributions.

[R17] Kermack WO, McKendrick A (1927). A contribution to the mathematical theory of epidemics. Proc R Soc Lond.

[R18] Khan ZS, Van Bussel F, Hussain F (2020). A predictive model for Covid-19 spread - with application to eight US states and how to end the pandemic. Epidemiol Infect.

[R19] Lam Y (1988). Geometric process and replacement problem. Acta Mathematica Appl Sinica.

[R20] Müller S, Regensburger G (2012). Generalized mass action systems: Complex balancing equilibria and sign vectors of the stoichiometric and kinetic-order subspaces. SIAM J Appl Math.

[R21] Nature (2021). Nature wades through the literature on the new coronavirus - and summarizes key papers as they appear. Nature.

[R22] Peliti L (2011). Statistical Mechanics in a Nutshell.

[R23] Press W, Flannery B, Teukolsky S, Vetterling W (2007). Numerical Recipes.

[R24] Roser M, Ritchie H, Ortiz-Ospina E, Hasell J (2021). Coronavirus pandemic (covid-19).

[R25] Singh RK, Rani M, Bhagavathula AS, Sah R, Rodriguez-Morales AJ, Kalita H (2020). Prediction of the covid-19 pandemic for the top 15 affected countries: Advanced autoregressive integrated moving average (arima) model. JMIR Public Health Surveill.

[R26] Weiss H (2013). The SIR model and the foundations of public health. MatMat.

[R27] Wenbin W, Ziniu W, Chunfeng W, Hu R (2013). Modelling the spreading rate of controlled communicable epidemics through an entropy-based thermodynamic model. Sci China Ser G Phys Mech Astron.

[R28] Yang C, Wang J (2020). A mathematical model for the novel coronavirus epidemic in wuhan, china. Math Biosciences Eng.

