# Supplementary material for: Monitoring and Forecasting COVID-19: Heuristic Regression, Susceptible-Infected-Removed Model and, Spatial Stochastic
Source: Front Appl Math Stat. Author manuscript; Available in PMC 2021 Jul 29. (PMC7611421; doi:10.3389/fams.2021.650716)
Supplement: SI [file EMS130929-supplement-SI.PDF]

Supplementary Material

1 MAIN PUBLIC-HEALTH ACTIONS SUGGESTED BY THE MODELS.

As explained in the main text, the generalized SIR model is summarized by the set of differential equations:

$$\frac{\mathrm{d}S(t)}{\mathrm{d}t} = -\frac{(S(t))^n}{\tau_0}\, i(t) \tag{S1}$$

$$\frac{\mathrm{d}I}{\mathrm{d}t} = \frac{(S(t))^n}{\tau_0}\, i(t) - \frac{I(t)}{\tau_1} \tag{S2}$$

$$\frac{\mathrm{d}R}{\mathrm{d}t} = \frac{(I(t))}{\tau_1} \tag{S3}$$

We summarize a few known public-health actions suggested by the SIR model (Weiss, 2013) which are useful to follow the lines of reasoning developped in the paper.

1. Since the maximum value for $R(\infty)$ is the entire population, $N < \infty$, the disease always dies out, $I(t > t_0) = 0$. Otherwise, if for some initial conditions we could have $I(\infty) \neq 0$, Eq. S3 would imply that $R(t)$ could grow without limit since $\frac{\mathrm{d}R}{\mathrm{d}t} > 0$, which proves the fact by *reductio ad absurdum*.
2. For $n = 1$ the ratio $\Re_0 = \frac{\tau_1}{\tau_0}$ determines whether the disease grows or dies. Since $S(t)$ can only decrease, we have from Eq. S2 that

$$\frac{\mathrm{d}I}{\mathrm{d}t} = \frac{S(t)\, i(t)}{\tau_0} - \frac{I(t)}{\tau_1} \leq \frac{1}{\tau_1}\left(\frac{\tau_1}{\tau_0} s(0) - 1\right) I(t) = \frac{1}{\tau_1}\left(\Re_0 - 1\right) I(t) = \frac{I(t)}{\tau} \tag{S4}$$

   at the onset, for $\Re_0 >> 1$, an initial estimation for τ_0 can be obtained from $\tau \approx \tau_0$. [1] Moreover, if $\Re_0 < 1$, $I(t)$ is a monotonically decreasing function and the infection dies quickly. On the other hand, if $\Re_0 > 1$, $I(t)$ increases in the region near $t = 0$, it reaches a maximum value, $I_M(t_M)$, and then it goes to zero, as proved in the point above. $\Re_0$ is called the **basic reproductive number** and it sets up a non-obvious threshold for the expansion of the disease.
3. For $n = 1$, the maximum number of infected people can be obtained by dividing the two equations S1 and S2,

$$\frac{\mathrm{d}S}{\mathrm{d}I} = -\frac{s(t)\; I(t)}{\frac{s(t)\; I(t)}{\tau_0} - \frac{I(t)}{\tau_1}} \tag{S5}$$

   which can be integrated to yield for $s(0) \approx 1$ and $i(0) \approx \frac{1}{N}$,

$$i_M = 1 - \frac{1}{\Re_0}\left(1 + \ln \Re_0\right) \tag{S6}$$

[1] $s(0) \approx 1$ for large N.

4. Similarly, dividing the equation S1 by S3 ($n = 1$), we get

$$\frac{\mathrm{d}S}{\mathrm{d}R} = -\Re_0 S \tag{S7}$$

i.e., $S(t) = S(0)e^{-\Re_0 R(t)}$, assuming $R(0) = 0$. Notice that for $\Re_0 >> 1$, $S(\infty)$ might get to the value zero, which corresponds to a very virulent epidemics where everybody dies.

The above considerations yield to the following public-health actions while dealing with an infectious disease (Figs. S1 and S2):

1. Reduce the contact rate or transmissibility, $\frac{1}{\tau_0}$, by isolating infectious nodes, encouraging frequent hand washing and the use of face masks. **Increasing values of τ_0 displace t_M towards larger times and decrease the value of I_M.**
2. Decrease τ_1 to reduce the duration of infection. **Increasing values of τ_1 displace t_M towards the origin and increase the value of I_M.**
3. Reduce $N = S(0)$ by vaccination or any other kind of immunity. **Increasing $S(0)$ displaces t_M towards larger values, decreases i_M and r_M..**
4. Decrease n. **Decreasing $n < 1$ moves t_M towards larger values and, it decreases i_M and r_M.**

REFERENCES

Weiss, H. (2013). The SIR model and the foundations of public health. *MatMat* 3, 1–17

2 SUPPLEMENTARY TABLES AND FIGURES

2.1 Figures

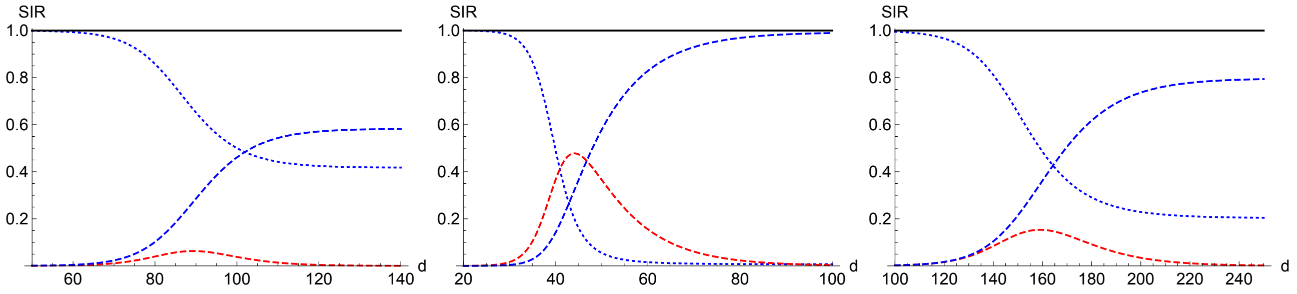

Figure S1. Representative behaviour of the SIR model ($n = 1$) depending on the parameters ($\Re_0 \geq 1.5$). Blue dotted: $S(t)$. Red dotted: $I(t)$. Blue dashed: $R(t)$. Initial conditions $N = 10000000 = S(0) + 1$, $I(0) = 1$. Left to right: (I) $\tau_1 = 2$, $\tau_0 = 3$, $t_M = 90$, $r(\infty) = 0.58$; (II) $\tau_1 = 2$, $\tau_0 = 10$, $t_M = 44$, $r(\infty) = 0.99$; (III) $\tau_1 = 5$, $\tau_0 = 10$, $t_M = 159$, $r(\infty) = 0.80$.

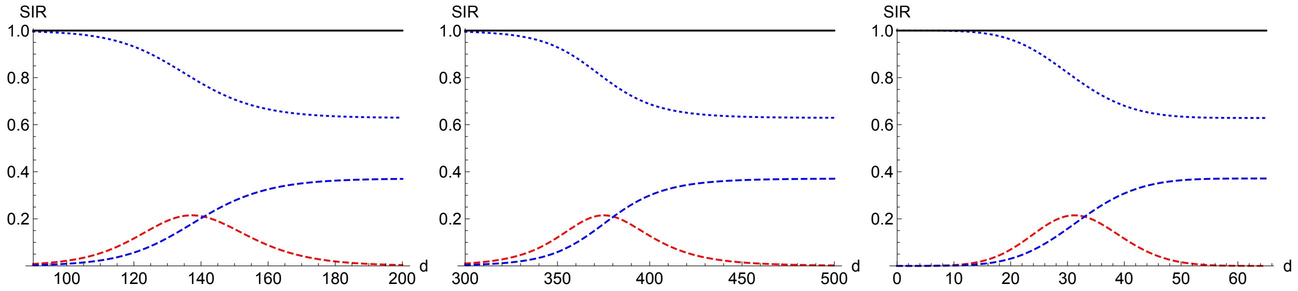

Figure S2. Representative behaviour of the SIR model ($n \neq 1$) depending on the parameters ($\Re_0 \leq 1.5$). Blue dotted: $S(t)$. Red dotted: $I(t)$. Blue dashed: $R(t)$. Initial conditions: $N = 10000000 = S(0) + 1$, $I(0) = 1$. Parameters: $\tau_1 = 2.5$, $\tau_0 = 2$; $\Re_0 = 1.25$, $r(\infty) = 0.37$. Left to right: (I) $n = 1$, $t_M = 137$; (II) $n = 1.1$, $t_M = 375$; (III) $n = 0.818$, $t_M = 31$.
